# Association between the Serum Soluble Urokinase Plasminogen Activator Receptor and Peripheral Arterial Stiffness According to the Cardio-Ankle Vascular Index in Patients Undergoing Kidney Transplantation

**DOI:** 10.31083/j.rcm2506219

**Published:** 2024-06-17

**Authors:** Hsiao-Hui Yang, Yen-Cheng Chen, Ching-Chun Ho, Bang-Gee Hsu

**Affiliations:** ^1^Department of Surgery, Hualien Tzu Chi Hospital, Buddhist Tzu Chi Medical Foundation, 97004 Hualien, Taiwan; ^2^School of Medicine, Tzu Chi University, 97004 Hualien, Taiwan; ^3^Division of Nephrology, Hualien Tzu Chi Hospital, Buddhist Tzu Chi Medical Foundation, 97004 Hualien, Taiwan

**Keywords:** cardio-ankle vascular index, kidney transplantation, peripheral arterial stiffness, soluble urokinase plasminogen activator receptor

## Abstract

**Background:**

High soluble urokinase plasminogen activator receptor (suPAR) levels are correlated with cardiovascular (CV) disease. Arterial stiffness is associated with aging-related vascular diseases and is an independent risk factor for CV morbidity and mortality. It can be measured by the cardio-ankle vascular index (CAVI). We evaluated the association between serum suPAR levels and arterial stiffness according to the CAVI in kidney transplantation (KT) recipients.

**Methods:**

In this study, 82 patients undergoing KT were enrolled. Serum suPAR levels were analyzed using an enzyme immunoassay. The CAVI was measured using a plethysmograph waveform device, and patients with a CAVI of ≥9.0 were assigned to the peripheral arterial stiffness (PAS) group.

**Results:**

Twenty KT patients (24.4%) had PAS, were of older age (*p* = 0.042), and had higher serum triglyceride (*p* = 0.023) and suPAR levels (*p* < 0.001) than the normal group. After adjusting for factors significantly associated with PAS by multivariate logistic regression analysis, serum suPAR levels (odds ratio [OR] 1.072, 95% confidence interval (CI) 1.023–1.123; *p* = 0.004) were independently associated with PAS in KT patients. The logarithmically transformed suPAR level (log-suPAR) was also positively correlated with the left or right CAVI values (all *p* < 0.001) from the results of the Spearman correlation analysis in KT patients.

**Conclusions:**

Serum suPAR levels are positively associated with left or right CAVI values and are independently associated with PAS in KT patients.

## 1. Introduction

Cardiovascular disease (CVD) remains one of the significant causes of mortality in kidney transplantation (KT) recipients with a functioning allograft. The preexisting risk factors for CVD in KT recipients are aggravated by post-transplantation immunosuppressive agents, obesity, diabetes mellitus, hypertension, and dyslipidemia [1]. Determining the estimated risk of CVD to identify KT recipients at risk of cardiovascular events is important to improve survival, and graft outcomes [2]. Arterial stiffness is a significant predictor of cardiovascular events, and several indices have been proposed to measure arterial stiffness, including the carotid-femoral pulse-wave velocity (PWV), brachial-ankle PWV, and cardio-ankle vascular index (CAVI) [3]. Among them, the CAVI is obtained automatically by wrapping pressure cuffs around the upper arms and lower legs, making it a noninvasive indicator of peripheral arterial stiffness (PAS) from the origin of the ascending aorta to the ankle [4]. The CAVI is clinically helpful in stratifying patients with atherosclerotic risk factors, and a higher risk of cardiovascular events was reported in patients with a higher CAVI [5, 6].

The soluble urokinase plasminogen activator receptor (suPAR), a key player in inflammation and fibrinolysis, has emerged as a predictive marker for CVD and atherosclerosis development [7, 8, 9]. Previous studies have established a link between suPAR levels and chronic kidney disease (CKD), with high suPAR levels robustly predicting all-cause and cardiovascular mortality in a large hemodialysis population in Italy [10, 11]. In a prospective study of KT recipients, suPAR levels significantly dropped after resolving the end-stage renal disease status. They were an early marker for allograft dysfunction during the follow-up period, highlighting its causal and prognostic role in CKD. Recently, the influence of suPAR on predicting cardiovascular events and mortality was investigated for the first time in KT recipients [12]. The study highlighted cardiovascular death as the leading cause of mortality, with patients exhibiting high suPAR levels having a quadrupled risk. Our research aims to elucidate the relationship between serum suPAR levels and PAS measured by the CAVI in KT recipients. This could establish suPAR as an innovative risk stratification biomarker and guide targeted interventions to prevent or mitigate the progression of arterial stiffness and CVD in post-KT care.

## 2. Materials and Methods

### 2.1 Patients

This cross-sectional study was conducted at a medical center in Hualien, Taiwan, recruiting 82 KT recipients between December 1, 2021 and June 30, 2022. Before participating, participants were briefed on the study’s purpose, were older than 18 years old, had a life expectancy of more than 6 months, had KT vintage of more than 6 months since KT, and obtained informed consent. The initial demographics, medication regimen, and relevant medical history were analyzed. Information on immunosuppressive agents, such as tacrolimus, cyclosporine, mycophenolate mofetil, rapamycin, and steroids, was collected through the patients’ medical records. The use of antihypertensive medications defined hypertension, while diabetes mellitus was recognized either through medical history or through the prescription of antidiabetic medications. Exclusion criteria specified individuals with a dialysis fistula or grafts, those with acute infections, acute rejection, malignancy, congestive heart failure (defined by the Framingham Diagnostic Criteria for Heart Failure) [13], and those who declined to consent to the research. The ethical oversight for this investigation was provided by the Hualien Tzu Chi Hospital Research Ethics Committee under the Buddhist Tzu Chi Medical Foundation (IRB108-219-A), ensuring compliance with the ethical guidelines outlined in the Declaration of Helsinki.

### 2.2 Anthropometric Analysis and Biochemical Investigations

To calculate the body mass index (BMI), the formula used was the individual’s weight in kilograms (kg) divided by their height in meters squared ($m^{2}$). Participants underwent an 8-hour fast before collecting a 5-mL blood specimen which was immediately centrifuged at 3000 ×*g* for 10 min. The analysis of serum fasting glucose, total cholesterol, triglyceride (TG), high-density lipoprotein cholesterol (HDL-C), low-density lipoprotein cholesterol (LDL-C), blood urea nitrogen (BUN), creatinine, calcium, and phosphorus levels was conducted using a Siemens Advia 1800 autoanalyzer (Siemens Advia 1800, Siemens Healthcare GmbH, Henkestr, Erlangen, Germany) [14]. The estimated glomerular filtration rate (eGFR) was calculated using the Chronic Kidney Disease Epidemiology Collaboration 2021 equation. Additionally, the levels of serum suPAR were measured with an enzyme immunoassay kit provided by Cloud-Clone Corp (Katy, TX, USA), and intact parathyroid hormone (iPTH) levels were assessed using an enzyme-linked immunosorbent assay kit from IBL International GmbH (Hamburg, Germany) [15].

### 2.3 Blood Pressure and CAVI Measurements

After blood sampling, the patients were instructed to rest in the supine position for 10 minutes within a calm environment where the temperature was regulated. Blood pressure measurements were obtained using an automatic oscillometric blood pressure monitor, recording systolic and diastolic pressures three times at the right brachial artery. The CAVI value was assessed using a previously outlined technique using the VaSera VS-1000 device (Fukuda Denshi Co. Ltd., Tokyo, Japan) [16]. During the CAVI measurement, the participant remained supine with the head aligned to the center, and cuffs were secured around both the upper arms and ankles. Phonocardiography microphones and electrocardiography electrodes were also used. VaSera VS-1000 was used to measure blood pressure and pulse wave velocity, and the CAVI value was automatically calculated. Patients with a CAVI value of ≥9.0 were considered to have PAS, based on the consensus of the Vascular Failure Committee of the Japan Society for Vascular Failure [17, 18]. For this research, participants were categorized into the PAS group if their CAVI value was 9.0 or higher, whereas those with CAVI values below 9.0 were placed in the control group.

### 2.4 Statistical Analyses

The distribution of the data was evaluated for normalcy using the Kolmogorov-Smirnov test. Data following a normal distribution are depicted as means ± standard deviations, with comparisons across patient groups conducted via the Student’s independent *t*-test (two-tailed). For data not adhering to a normal distribution, medians and interquartile ranges are provided, and the Mann-Whitney U test was utilized for comparisons, covering variables such as age, TG, fasting glucose, BUN, creatinine, iPTH, and suPAR levels. A logarithmic transformation (base 10) was applied for non-normally distributed variables to achieve normalcy before performing statistical comparisons. Categorical data are represented as counts and percentages, with the chi-square test applied for comparative analysis. Variables significantly associated with PAS were further evaluated using multivariate logistic regression analysis. Additionally, Spearman’s rank-order correlation coefficient was used to examine the association between log-transformed suPAR, left CAVI, right CAVI, and other variables. The receiver operating characteristic (ROC) curve analysis was performed to identify the log-suPAR level indicative of PAS among KT patients. Analysis was carried out using the Statistical Package for the Social Sciences (SPSS version 19.0, IBM Corp., Armonk, NY, USA), with a *p*-value of less than 0.05 indicating statistical significance.

## 3. Results

Table 1 shows the clinical characteristics of the 82 KT recipients included in this study. Among them, 28 patients had diabetes mellitus, and 34 patients had hypertension. Twenty patients (24.4%) were classified into the PAS group based on the CAVI results. Significantly more patients in the PAS group were older (*p* = 0.042). Furthermore, they exhibited higher serum triglyceride (*p* = 0.023) and suPAR (*p* < 0.001) levels than the control group. However, no significant differences in systolic and diastolic blood pressures, sex, KT duration, BMI, hypertension, living donor, and immunosuppressive drugs used were observed between the two groups.

**Table 1.** **Clinical variables of kidney transplantation patients with or without peripheral arterial stiffness**.

Characteristic	All participants (n = 82)	Normal CAVI group (n = 62)	High CAVI group (n = 20)	*p*-value
Age (years)	56.00 (47.75–62.00)	55.00 (42.75–62.00)	61.00 (54.00–62.00)	0.042*
KT vintage (months)	89.90 ± 60.16	91.43 ± 54.99	98.07 ± 59.97	0.592
Height (cm)	160.83 ± 10.79	159.89 ± 11.28	163.75 ± 8.70	0.165
Body weight (kg)	66.65 ± 15.55	67.45 ± 15.48	64.15 ± 15.89	0.413
Body mass index (kg/$m^{2}$)	25.23 ± 4.80	25.75 ± 4.93	23.64 ± 4.08	0.087
Left CAVI	7.30 ± 2.44	6.24 ± 1.33	10.62 ± 2.07	<0.001*
Right CAVI	7.35 ± 2.62	6.24 ± 1.33	10.81 ± 2.64	<0.001*
Systolic blood pressure (mmHg)	141.99 ± 17.70	140.73 ± 16.88	145.90 ± 19.97	0.258
Diastolic blood pressure (mmHg)	83.17 ± 11.36	83.55 ± 11.02	82.00 ± 12.60	0.599
Total cholesterol (mg/dL)	182.16 ± 41.29	179.27 ± 37.16	191.10 ± 52.18	0.268
Triglyceride (mg/dL)	134.00 (98.75–196.00)	122.00 (89.00–175.75)	176.50 (120.25–228.25)	0.023*
HDL-C (mg/dL)	52.01 ± 15.90	52.85 ± 14.00	49.40 ± 20.94	0.401
LDL-C (mg/dL)	101.20 ± 28.58	98.48 ± 25.05	109.60 ± 37.00	0.131
Fasting glucose (mg/dL)	94.00 (87.75–109.25)	94.00 (88.00–109.00)	93.50 (87.00–126.50)	0.983
Blood urea nitrogen (mg/dL)	25.50 (16.00–35.00)	25.00 (16.00–33.25)	26.50 (18.25–44.75)	0.280
Creatinine (mg/dL)	1.36 (1.08–1.96)	1.29 (1.02–1.72)	1.68 (1.12–2.25)	0.142
eGFR (mL/min)	53.17 ± 25.35	55.31 ± 25.37	46.55 ± 24.75	0.180
Total calcium (mg/dL)	9.39 ± 0.71	9.31 ± 0.72	9.42 ± 1.00	0.566
Phosphorus (mg/dL)	3.32 ± 0.74	3.29 ± 0.77	3.38 ± 0.75	0.584
iPTH (pg/mL)	81.70 (50.40–150.60)	85.75 (51.93–156.35)	76.80 (33.85–122.38)	0.578
suPAR (pg/mL)	58.50 (53.06–74.31)	55.89 (51.39–65.75)	89.46 (61.15–177.32)	<0.001*
Female, n (%)	40 (48.8)	31 (50.0)	9 (45.0)	0.697
Diabetes, n (%)	28 (34.1)	30 (32.3)	8 (40.0)	0.526
Hypertension, n (%)	34 (41.5)	24 (38.7)	10 (50.0)	0.513
Living donor, n (%)	25 (30.5)	19 (30.6)	6 (30.0)	0.957
Steroid use, n (%)	73 (89.0)	55 (88.7)	18 (90.0)	0.872
Cyclosporine use, n (%)	8 (9.8)	7 (11.3)	1 (5.0)	0.410
Tacrolimus use, n (%)	71 (86.6)	52 (83.9)	19 (95.0)	0.204
Mycophenolate mofetil use, n (%)	67 (81.7)	52 (83.9)	15 (75.0)	0.372
Statin use, n (%)	34 (41.5)	28 (45.2)	6 (30.0)	0.231
Fibrate use, n (%)	19 (23.2)	13 (21.0)	6 (30.0)	0.405

Values for continuous variables are shown as mean ± standard deviation after analysis by Student’s *t*-test; variables not normally distributed are shown as median and interquartile range after analysis by the Mann-Whitney U test; values are presented as number (%) and analysis after analysis by the chi-square test. KT, kidney transplantation; CAVI, cardio-ankle vascular index; HDL-C, high-density lipoprotein cholesterol; LDL-C, low-density lipoprotein cholesterol; eGFR, estimated glomerular filtration rate; iPTH, intact parathyroid hormone; suPAR, soluble urokinase-type plasminogen activator receptor. * *p* < 0.05 was considered statistically significant.

**Fig. 1.**
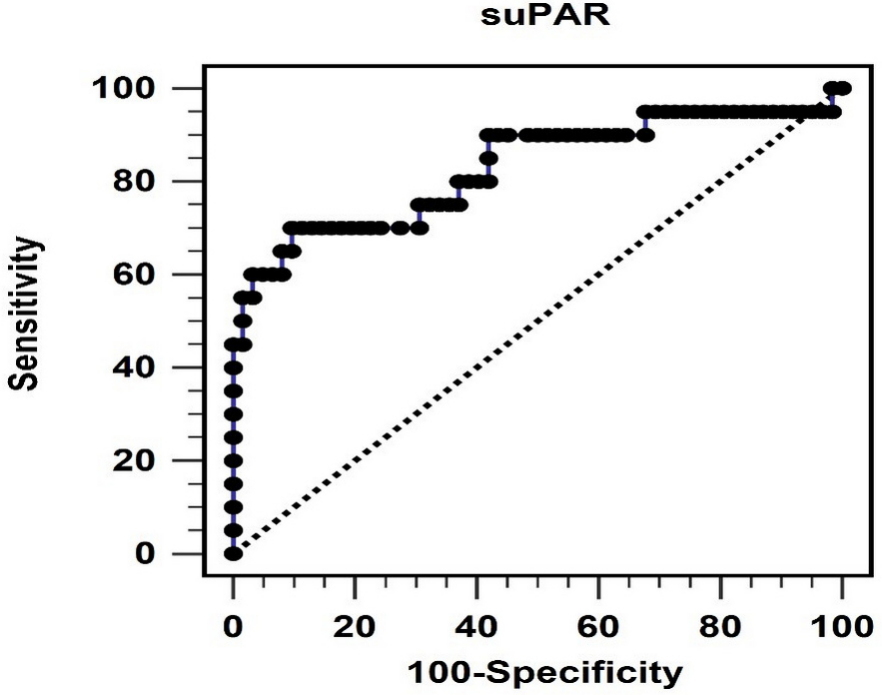
**The area under the receiver operating characteristic curve indicates the diagnostic power of soluble urokinase-type plasminogen activator receptor levels for predicting peripheral arterial stiffness. **suPAR, soluble urokinase-type plasminogen activator receptor.

After adjusting for factors that showed an association with PAS (i.e., age, triglyceride, BMI, LDL-C, eGFR, and suPAR from Table 1; *p* < 0.2), suPAR emerged as a significant predictor of PAS in patients with KT (odds ratio [OR]: 1.072; 95% confidence interval (CI): 1.023–1.123; *p* = 0.004) after multivariate logistic regression analysis, as shown in Table 2. The ROC curve analysis for predicting PAS using suPAR yielded an area under the ROC curve (AUC) of 0.829 (95% CI, 0.730–0.903; *p* < 0.001) (Fig. 1). According to the Youden index, the optimal cutoff suPAR level for predicting PAS was 73.97 pg/mL (sensitivity: 70.0%; specificity: 90.32%; positive predictive value: 70.01%; and negative predictive value: 90.32%).

**Table 2.** **Multivariable logistic regression analysis of the factors correlated to peripheral arterial stiffness**.

Variables	Odds ratio	95% confidence interval	*p*-value
suPAR, 1 pg/mL	1.072	1.023–1.123	0.004*
Age, 1 year	1.098	0.996–1.209	0.060
Triglyceride, 1 mg/dL	1.009	0.997–1.020	0.130
Body mass index, 1 kg/$m^{2}$	0.801	0.635–1.010	0.060
LDL-C, 1 mg/dL	1.016	0.990–1.042	0.243
eGFR, 1 mL/min	1.000	0.972–1.028	0.977

Data was analyzed using the multivariate logistic regression analysis (adopted factors: age, triglyceride, body mass index, LDL-C, eGFR, and suPAR). LDL-C, low-density lipoprotein cholesterol; eGFR, estimated glomerular filtration rate; suPAR, soluble urokinase-type plasminogen activator receptor. * *p* < 0.05 was considered statistically significant.

Furthermore, we explored the correlation between log-suPAR, left CAVI, right CAVI, and other variables using Spearman’s rank order correlation coefficient, as shown in Table 3. Both the left and right CAVI values were positively associated with log-suPAR (all *p* < 0.001), and log-triglyceride levels were negatively associated with log-suPAR (*p* = 0.033). Furthermore, log-age was positively correlated with both the left CAVI (*p* = 0.014) and right CAVI (*p* = 0.007) values, and log-triglyceride levels were positively correlated with both the left CAVI (*p* = 0.040) and right CAVI (*p* = 0.033) values.

**Table 3.** **Spearman correlation coefficients between left CAVI, right CAVI, suPAR, and clinical variables in kidney transplantation patients**.

Variables	Left CAVI		Right CAVI		Log-suPAR (pg/mL)	
	Spearman’s coefficient of correlation	*p*-value	Spearman’s coefficient of correlation	*p*-value	Spearman’s coefficient of correlation	*p*-value
Left CAVI	**—**	**—**	0.898	<0.001*	0.659	<0.001*
Right CAVI	0.898	<0.001*	**—**	**—**	0.687	<0.001*
Log-suPAR (pg/mL)	0.659	<0.001*	0.687	<0.001*	**—**	**—**
Log-Age (years)	0.271	0.014*	0.294	0.007*	0.088	0.433
KT vintage (months)	–0.016	0.888	0.002	0.988	–0.211	0.058
SBP (mmHg)	0.150	0.180	0.153	0.170	0.217	0.050
DBP (mmHg)	–0.027	0.807	–0.015	0.894	0.123	0.270
Total cholesterol (mg/dL)	0.121	0.279	0.156	0.162	0.080	0.473
Log-Triglyceride (mg/dL)	0.228	0.040*	0.236	0.033*	–0.235	0.033*
HDL-C (mg/dL)	–0.079	0.480	–0.042	0.709	–0.107	0.338
LDL-C (mg/dL)	0.181	0.104	0.130	0.245	0.039	0.731
Log-Glucose (mg/dL)	0.110	0.326	0.157	0.159	0.126	0.258
eGFR (mL/min)	–0.150	0.179	–0.084	0.451	–0.129	0.433
Total calcium (mg/dL)	0.021	0.848	0.012	0.914	–0.005	0.965
Phosphorus (mg/dL)	–0.050	0.653	–0.097	0.388	–0.080	0.477
Log-iPTH (pg/mL)	0.018	0.870	–0.009	0.933	0.066	0.558

Data on suPAR, age, triglyceride, glucose, and iPTH levels showed a skewed distribution and were log-transformed before analysis. Data analysis was performed using Spearman correlation analysis. KT, kidney transplantation; CAVI, cardio-ankle vascular index; SBP, systolic blood pressure; DBP, diastolic blood pressure; HDL-C, high-density lipoprotein cholesterol; LDL-C, low-density lipoprotein cholesterol; eGFR, estimated glomerular filtration rate; suPAR, soluble urokinase-type plasminogen activator receptor; iPTH, intact parathyroid hormone. * *p* < 0.05 was considered statistically significant.

## 4. Discussion

Our study found that 24.4% of KT recipients exhibited PAS, as indicated by the CAVI results. These PAS recipients were significantly older and had higher serum TG and suPAR levels than the control group. Furthermore, we found that suPAR levels independently predicted PAS. Additionally, significant correlations were observed between log-suPAR, CAVI values, age, and TG levels, indicating their association with arterial stiffness.

Arterial stiffness reflects structural changes in the arterial wall associated with loss of elasticity and estimates the extent of atherosclerosis. It is considered a strong predictor of cardiovascular events and mortality and can be measured using the CAVI, which has demonstrated clinical efficacy [4, 5, 6]. For patients who have undergone KT, cardiovascular events significantly affect survival and graft outcomes [2]. Therefore, an available biomarker is required to detect and prevent atherosclerosis and CVD.

Age is associated with arterial stiffness in various groups of patients, including those with hypertension, diabetes mellitus, and metabolic syndrome [19]. Aging leads to changes in the elastin and collagen of the arteries and rearrangements of the extracellular matrix architecture, contributing to altered endothelial function and atherogenesis [20]. Yue *et al*. [21] found a linear increase in CAVI values with age in both sexes among patients with metabolic syndrome (*p* for trend <0.001) in the general Chinese population. Our study also observed that KT patients with PAS were significantly older. A significant positive association was observed between the CAVI and log-age, indicating that the CAVI values tend to increase as age increases.

Dyslipidemia is a well-established risk factor for the development and progression of CVD [22]. Although whether lipid parameters act as pathogenic mediators or markers of atherosclerosis remains unclear, we found that KT patients with PAS had significantly higher TG levels. Studies have shown contradictory results on the association between high TG levels and arterial stiffness. Yue *et al*. [21] reported no association between high TG levels and PAS measured using the CAVI in the general Chinese population. However, a random population-based study demonstrated a significant and positive correlation between TG and the CAVI, independent of known confounding factors, such as age, sex, metabolic syndrome components, LDL-C, statin use, and smoking status [23]. Another retrospective cross-sectional study involving 23,537 healthy Japanese residents revealed a cutoff value of 93 mg/dL for TG to predict high CAVI values (AUC = 0.735) [24]. Several studies conducted in China also reported a positive correlation between TG and brachial-ankle PWV, another measurement of arterial stiffness [25, 26]. Our analysis also demonstrated a positive correlation between the CAVI and log-TG levels, indicating that higher log-TG levels were associated with PAS. Furthermore, we did not find a significant correlation between other lipid profiles (e.g., total cholesterol, HDL-C, and LDL-C) and PAS.

suPAR is a plasma glycoprotein implicated as an independent risk marker for CVD, playing a direct role in atherogenesis and neointimal lesion formation [27]. The South African study regarding the role of Sex, Age and Ethnicity on Insulin sensitivity and Cardiovascular function (SAfrEIC) study first investigated the role of suPAR in measuring arterial stiffness among different ethnicities [28]. Further studies have found a positive correlation between suPAR and aortic PWV in patients with chronic obstructive pulmonary disease and type I diabetes mellitus [29, 30]. These conditions are systemic inflammatory diseases associated with an increased risk of CVD. Furthermore, a strong association was observed between suPAR levels and carotid-femoral PWV in a multivariate linear regression analysis involving hemodialysis patients [15]. In our study, suPAR was the only significant predictor of PAS in KT patients after multivariate logistic regression analysis. This underscores the potential of suPAR as a critical biomarker in predicting vascular complications post-transplantation. We also observed a positive correlation between the CAVI and log-suPAR levels. Additionally, log-TG levels were negatively correlated with log-suPAR levels. Recent research by Haupt *et al*. [31] further explores this relationship, demonstrating a strong positive correlation between TG and suPAR levels across a general population. However, this correlation dissipated upon conducting multiple linear regression analysis, indicating that other underlying factors might influence the relationship between TG levels and suPAR [31].

suPAR is an innate immune activator in acute kidney injury (AKI) and chronic kidney disease [32]. It interacts with integrins on podocytes, mediating the renal filtration barrier function and providing a molecular foundation for certain glomerular kidney diseases [7]. Hayek *et al*. [33] discovered that elevated suPAR levels were linked to AKI and mortality within 90 days among patients exposed to intra-arterial contrast for coronary angiography, those who underwent cardiac surgery, and those admitted to intensive care unit (ICU) for critical illness. The underlying mechanism involves suPAR sensitizing the kidney’s proximal tubules to injury by modulating cellular bioenergetics and increasing oxidative stress, leading to AKI [33]. In a broad, unselected hemodialysis cohort, a high suPAR level was a predictor of all-cause mortality (hazard ratio [HR] = 1.91, 95% CI = 1.47–2.48, *p* < 0.001), CV mortality (HR = 1.47, 95% CI = 1.03–2.09, *p* = 0.03), and non-CV mortality (HR = 1.94, 95% CI = 1.28–2.93, *p* = 0.002) [10]. A subsequent prospective study of 100 KT recipients demonstrated a significant decrease in suPAR levels post-transplant [11], with a strong correlation observed between suPAR levels at 1-year post-KT and eGFR loss, highlighting its potential for early detection of allograft dysfunction. However, in our study, the suPAR level negatively correlated with eGFR but lacked statistical significance. In addition, Morath *et al*. [12] indicated that suPAR levels predict all-cause and cardiovascular death in 1023 KT recipients, independent of transplantation timing and primary kidney disease. Cardiovascular death is the predominant cause of mortality, marked by a significant HR of 4.24 in patients with elevated suPAR levels. In our study, serum suPAR levels were positively correlated with CAVI values and independently associated with arterial stiffness, indicating a link to atherosclerosis and subsequent cardiovascular disease. suPAR could be instrumental in identifying KT recipients at an elevated risk of cardiovascular death, enabling optimized follow-up and post-transplant care.

This study has several limitations. This was a single-center cross-sectional study with a small sample size. Enrolling more participants and conducting longitudinal studies with an extended follow-up period may help strengthen the causal relationship between serum suPAR levels and PAS during the evolution of CVD. Furthermore, the clinical application of suPAR levels and the appearance of CVD in KT recipients require further investigation. Such studies may improve our understanding of the potential implications for clinical practice and guide the development of preventive and therapeutic strategies.

## 5. Conclusions

In conclusion, KT patients with PAS were significantly older and exhibited higher serum suPAR and TG levels. Serum suPAR levels are positively associated with left or right CAVI values and are independently associated with PAS in KT recipients. These insights pave the way for improved risk stratification and management strategies for KT recipients at risk of PAS, aiming to prevent cardiovascular events.

## Data Availability

The data presented in this study are available on request from the corresponding author.
